# Rehabilitation of Peripheral Neuropathies: From Lexical Analysis of the Literature to Identification of Clinical Protocols

**DOI:** 10.3390/jcm12185879

**Published:** 2023-09-10

**Authors:** Daniele Coraci, Marcello Romano, Lisa Ragazzo, Domenico Antonio Restivo, Martina Cipriani, Federica Gottardello, Martina Pizzolato, Maria Chiara Maccarone, Stefano Masiero

**Affiliations:** 1 Department of Neuroscience, Section of Rehabilitation, University of Padova, Via Nicolò Giustiniani, 2, 35128 Padua, Italy; lisa.ragazzo@unipd.it (L.R.); martina.cipriani@studenti.unipd.it (M.C.); federica.gottardello@studenti.unipd.it (F.G.); martina.pizzolato@studenti.unipd.it (M.P.); stef.masiero@unipd.it (S.M.); 2 Neurology Unit, Azienda Ospedaliera Ospedali Riuniti Villa Sofia Cervello, 90146 Palermo, Italy; mc.romano1958@gmail.com; 3 Department of Clinical and Experimental Medicine, University of Messina, Piazza Pugliatti, 1, 98122 Messina, Italy; domenicoantonio.restivo@unime.it; 4 Physical Medicine and Rehabilitation School, University of Padova, Via Nicolò Giustiniani, 2, 35128 Padua, Italy; mariachiara.maccarone@phd.unipd.it

**Keywords:** peripheral nerve, neuropathies, rehabilitation, graph-theory

## Abstract

Peripheral nervous system diseases are a complex and heterogenous group of diseases affecting the different nerves with various severity and impact on quality of life. The current literature does not provide a structured guide for the rehabilitation of these conditions. We performed a lexical literature evaluation based on graph theory to clarify this topic. We performed a search on PubMed and calculated the frequencies of the words indicating rehabilitation approaches, nerves, and diseases. We found the usefulness of exercises and different physical agents, like laser and ultrasound therapy and neuromuscular stimulation vibration therapy. Orthoses are useful for entrapment, trauma, and hereditary diseases. Correct knowledge and assessment of the neuropathies are fundamental for the therapeutic decision and to guide rehabilitation. Despite the usefulness shown by the different approaches to modulating pain, improving muscle strength and endurance, and ameliorating balance and the sensory system, further studies are needed to define the best-personalized protocols.

## 1. Introduction

Peripheral nervous system diseases (PNDs) are a heterogeneous group of clinical conditions involving the peripheral nerves in potentially every part of the body [1]. These conditions present different etiologies and incidences; they are spread worldwide, and they cause functional impairments and disabilities with a high impact on quality of life (QoL) [2]. In this sense, PNDs represent one of the study objects of rehabilitation medicine, with a wide range of interventions but many challenges. The severity of each PND is mainly related to the etiopathogenesis, the localization, the type of nerve fibers involved (sensory, motor, or autonomic), and, more importantly, the difference between demyelinating and axonal forms [3,4,5]. Although in some cases mixed forms may be present, the demyelinating form represents specific damage to the myelin sheath constituted by the Schwann cells, the axonal form indicates specific damage to the axons forming the nerve. In demyelinating PNDs, the damaged structures are responsible for the “velocity”, so the lesion causes a slowdown of the electrical signals of the nerve; in the axonal form, the number of structures that transport the signals is lowered [6,7]. Although the symptoms in both forms may be similar, the underlying situation is totally different from the potentiality of evolution. This is particularly valid in cases of demyelinating motor neuropathies: in early phases and not extremely severe conditions, the muscle fibers and the muscular architecture are preserved because they depend on the integrity of the nerve axons [8]. In these neuropathies, the damaged myelin can be ideally restored if the cause of the disease is treated. On the other hand, in axonal motor neuropathies, the muscle fibers can lose their integrity. After an axonal lesion, the nerve goes through Wallerian degeneration, and this process is naturally followed by a regeneration of the axons inside the nerve if the myelin sheath is still intact. In motor axon involvement, the muscle fibers deprived of their nerve fibers can be innervated by the remaining axons through the mechanism of collateral sprouting [9]. This distinction may have a significant impact on rehabilitation management. Rehabilitation cannot modify the mechanisms of restoration but can facilitate them and support the patient in the disease phases.

Considering the localization, we can distinguish mononeuropathies, multineuropathies, and polyneuropathies [10]. The mononeuropathies include entrapment neuropathies, which represent a very common form mainly characterized by localized demyelinating damage in the compression site. When compression is particularly strong, chronic axonal damage can occur. However, compression neuropathies can represent the initial presentation of other PND forms, like genetic or systemic [11]. Nerve tumors, like schwannoma and neurofibroma, can be particularly impactful and can cause severe sensory and/or motor symptoms; they belong to the category of mononeuropathies, even if the involvement may be in multiple nerves in genetic conditions [12]. A particular category of mononeuropathies includes traumatic nerve injuries [13]. They occur after trauma and usually involve different tissues and organs, like bones, muscles, vessels, and nerves. In these cases, the trauma causes multiple damages to the nerve, disrupting the axons, myelin sheath, nerve stroma, and vasa nervorum. In these injuries, the distinction between axonotmesis (axonal interruption with nerve continuity preserved) and neurotmesis (nerve discontinuity) is fundamental because it is one of the factors determining the necessity of a surgical approach since regeneration along the nerve is impossible in neurotmesis [14]. Finally, among the mononeuropathies, immune-mediated neuropathies can be included. They are rarer forms in which an auto-immune reaction happens when the immune system produces an exaggerated response after a stimulus, like infection, stress, vaccination, or therapy [15]. Different nerves in different body parts can be involved, usually in asymmetric and irregular localization, thereby showing a multi-neuropathic presentation. Differently from multineuropathies, in polyneuropathies, the localization is usually symmetrical, with a relatively diffuse distribution and a proximal-distant gradient of severity. Many PNDs are included in this category, and they can have several etiopathogeneses. The most common immune-mediated neuropathies are demyelinating forms, distinguished between acute (like Guillain–Barré syndrome (GBS)) and chronic (like chronic demyelinating polyradiculoneuropathies (CIDP)). These forms can be particularly drastic because the severe diffuse demyelinating damage causes severe weakness, and, in chronic forms, the sequelae are visible with the common presentation of fatigue [16]. Among the axonal polyneuropathies, the toxic neuropathies represent a typical form. They are caused by the side effects of specific drugs (like chemotherapies) or by the effects of abused substances (like ethanol) [17,18]. Genetic polyneuropathies represent a vast category and can be both demyelinating and axonal [19]. Finally, the most common polyneuropathy is diabetic neuropathy; chronic hyperglycemia causes a wide spectrum of damages, including effects on the myelin and axons [20].

In addition to the complex classification of PNDs, the clinical presentation is not always clear, and the symptoms and their severity can be variegated. In cases of sensory involvement, neuropathic pain, allodynia, hypoesthesia, and sometimes itch may be present [21]. In motor involvement, a strength deficit and difficulties in motor tasks are present [22]. Autonomic disturbances may be complained about [23]. In general, the patients report a reduction in their abilities in activities of daily living and an impairment in QoL, especially when pain is present. Clinical evaluation is fundamental for the first diagnostic process [24]. However, the application of electrodiagnostic examination (EDx), imaging (especially ultrasound and magnetic resonance), lab tests, genetic tests, and nerve or skin biopsy allows the diagnostic definition and the therapeutic decision [25,26,27]. Treatment can be divided into surgical and conservative [28]. Surgery is mandatory in cases of neurotmesis, severe compressions, tumors, and if functional corrections are not possible in alternative ways (for example, tendon elongation or transposition). Conservative treatments include disease-specific drugs, pain treatments, and other non-specific drugs. At present, immune-mediated neuropathies are treatable with immunosuppressant and immunomodulating drugs (steroids, immunoglobulins, monoclonal antibodies, plasmapheresis), while only some genetic neuropathies have shown a good response to specific drugs [29]. No efficient drug is still available for the most common genetic neuropathy, Charcot–Marie–Tooth disease (CMT) [30]. For this disease, the intrinsic damage to myelin or axons cannot be avoided, so tissue restoration is not possible. For this reason, rehabilitation represents the main weapon against CMT. The treatment of neuropathic pain is one of the major targets in the management of PNDs because of its consequences in daily life. Antidepressants and anticonvulsants are the first-line approaches, followed by opioids in non-responsive cases [31]. A vast number of products exist for supporting the management of PNDs, including vitamins, neurotrophic factors, and nutraceuticals [32]. They are not disease-specific, but they have shown usefulness, especially as adjuvants in pain treatment, as well as in some non-conventional therapies like acupuncture [33].

Conservative treatments include rehabilitation programs, which have multiple aims: increase structural restoration, ameliorate function, prevent abnormal compensation, prevent or treat pain, and improve QoL. The rehabilitation aims are guided by the International Classification of Function (ICF), which allows a classification of body and function alterations and their associations with social activity and participation [34]. To reach these aims, rehabilitation uses multiple means able to act at different levels. In order to directly intervene in the neuromuscular structures and functions, different kinds of electromagnetic stimulation exist: neuromuscular electrical stimulation, which can avoid muscle atrophy and facilitate axonal sprouting; transcutaneous electrical nerve stimulation (TENS), able to modulate nerve signals; laser therapy; and capacitive and resistive electric transfer therapy, which are recognized to have anti-inflammatory and regeneration effects [35,36]. Other physical agents are applicable to treat pain and improve tissue and function restoration: ultrasound therapy and vibration therapy, based on mechanical waves; cryotherapy; and other thermal therapies [36]. Exercises and motor education represent a powerful method because they allow the patient to manage his/her energy resources, avoid abnormal gestures, and acquire strategies useful for daily activities. Differently from an antiquated vision, exercise is considered particularly important to treat fatigue and can aid the training of proprioception and sensory feedback [37]. Different types of manual therapies are also available for this latter aim and even to treat muscle contractures.

The mentioned rehabilitation approaches are not specific for each neuropathy, and, at the moment, a real identification of the most appropriate programs is not available. We performed this literature review to assess the current scientific knowledge about rehabilitation and PNDs. The aims of this review are: (1) evaluation of papers based on lexical analysis to show the relations of the words used in the papers to describe the various arguments; and (2) identification, on the basis of the current literature, of possible clinical protocols for rehabilitation of PNDs.

## 2. Materials and Methods

For literature analysis, we have used PubMed and the following string: “rehabilitation AND peripheral neuropath* AND (“physical agent” OR “physical therapy” OR electrotherapy OR “laser therapy” OR magnetotherapy OR “transcutaneous electrical nerve stimulation” OR “ultrasound therapy” OR “extracorporeal shock wave therapy” OR “capacitive and resistive electric transfer therapy” OR “electrical stimulation” OR “vibration therapy” OR “extracorporeal shock wave therapy” OR “cryotherapy” OR “thermal therapy” OR exercise OR massage OR manipulation OR mobilization)”. The words were chosen on the basis of the experience of the authors and the availability of rehabilitation approaches routinely applied in clinical practice. The terms were linked by the Boolean operators AND and OR to define and merge the different subjects. In the string, terms identifying the common physical agents and the usual manual treatment are included. We did not use the MeSH terms in order to obtain as many results as possible. Additionally, we used the following filters: article types (Clinical Trial, Guideline, Meta-Analysis, Randomized Controlled Trial, Review, Systematic Review), and publication date (the last 10 years). The found results were exported by the in-built function of the database in a text file containing the titles, the abstracts, and the editorial information. This file was analyzed to calculate the frequency of the words from a list including the diseases, the commonly assessed nerves, and the rehabilitation techniques. The words assessed were: trauma, injury, entrapment, compression, tumor, schwannoma, neurofibroma, hereditary, inherited, Charcot–Marie–Tooth, CMT, dysimmune, immune, Guillain–Barré, GBS, polyradiculoneuropathy, CIDP, diabetic, toxic; facial district, median, ulnar, radial, peroneal, tibial, sural, femoral nerves; rehabilitation, physical agent, physical therapy, exercise, manipulation, mobilization, electrotherapy, laser therapy, magnetotherapy, ultrasound therapy, capacitive and resistive electric transfer therapy, electromagnetic stimulation, vibration therapy, extracorporeal shock wave therapy, cryotherapy, thermal therapy, and orthosis. For each time a word was present in the title or the abstract of a paper, we assigned to the relationship word-paper a value of 1, or alternatively, we assigned a value of 0. Thus, we built a rectangular matrix with the paper on one side and the words on the other side. This matrix contained the binary values 0 or 1, as above described. It was imported into the freeware software Gephi 0.10.0, which is able to process, visualize, and analyze large networks based on graph theory. The described method, a lexical network based on graph theory (LENGTH), allows the evaluation of the connections between the papers and the words, transforming them into nodes connected by the edges [31,38]. The software permits the visualization of the network in different manners and can show the frequency of a word by the degree of a node, which can be converted into the respective largeness. In this way, the most frequent words show the largest dimension of the correspondent node. Through the software, specific statistics are possible; in particular, the recognition of classes (that are families of very connected papers and words) is possible by the calculation of modularity class, which distinguishes each family with a number [38].

To complete the literature evaluation, we have performed additional separate research on PubMed using the term “rehabilitation” linked by the operator AND to a word indicating a nerve or a disease. The results were exported and analyzed as described above. We normalized the frequency of the words for the number of papers for each nerve or disease, and we collected the data about the three most relatively frequent rehabilitation approaches for each nerve and each disease and the most relatively common nerve and disease for each rehabilitation approach. The results were summarized in a proper table.

Finally, on the basis of the collected information, we developed a flowchart to recapitulate the possible clinical protocols compatible with the current literature.

## 3. Results

### 3.1. General Results

The search strategy initially found 345 papers. After the analysis of the titles and the abstracts, a total of 317 papers were included in the network. Among the words initially considered, only 35 were present: trauma, injury, entrapment, compression, tumor, hereditary, inherited, Charcot–Marie–Tooth, CMT, immune, Guillain–Barré, polyradiculoneuropathy, diabetic, toxic; facial, median, ulnar, radial, peroneal, tibial, sural, femoral; rehabilitation, physical agent, physical therapy, exercise, manipulation, mobilization, electrotherapy, laser therapy, ultrasound therapy, stimulation, vibration therapy, cryotherapy, and orthosis.

Among the first list of rehabilitation approaches, no papers containing magnetotherapy, capacitive and resistive electric transfer therapy, extracorporeal shock wave therapy, or thermal therapy were found.

### 3.2. Lexical Network Based on Graph-Theory

Among the diseases, diabetes was the most common one, being present 254 times and in 98 papers, followed by the terms injury, toxic, compression, and immune, respectively, with a frequency of 111, 51, and 22 for compression and immune. They are present in 50, 29, 12, and 14 papers. Trauma and entrapment showed a frequency of 17 and 14, and they were present in 14 and 11 papers, respectively. The remaining diseases were mentioned less than 10 times. Considering the anatomical sites, the facial district, median, and ulnar nerves showed the highest occurrence, respectively 24, 18, and 14 times in 10, 8, and 5 papers. The radial nerve and the lower limb nerves presented a frequency from 4 to 10 times. Concerning rehabilitation approaches, exercise was the most common term and absolutely the word with the highest frequency, with a degree value of 507 and a presence in 122 papers. A high value was visible for stimulation, indicating the various forms of electromagnetic stimulation (TENS, peripheral current or magnetic stimulation, non-invasive brain stimulation, etc.). This term was used 375 times in 117 papers. Rehabilitation and physical therapy showed a frequency of 151 and 63 times, respectively. The other words indicating rehabilitation treatments were present less than 10 times.

**Figure 1:**
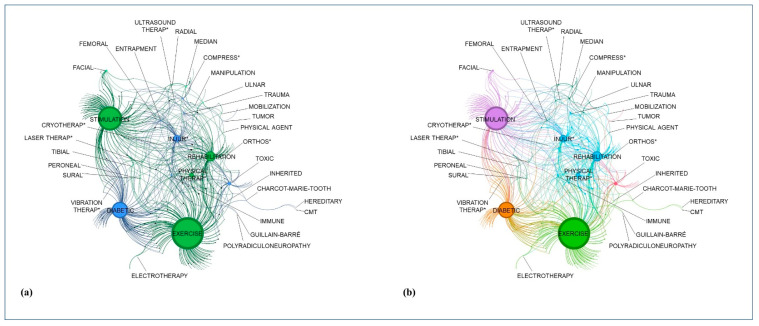
Results of the lexical network based on graph theory. (**a**) Presentation of the three main topics: rehabilitation treatment in dark green, nerves in light green, and diseases in blue; (**b**) Presentation of the six classes (each color indicates a class containing words and papers). The asterisks (*) indicates the possible terminal parts of the terms with the same word root (for example, compress-ed or compress-ion).

In the network, the dimension of the papers was usually small because a paper could have only limited connections with the words, while a word could be contained in many papers. Additionally, some papers were visible on the periphery of the network, thus indicating they have only a connection with a single word. In a similar way, a few words were visible in the periphery (hereditary, CMT, Guillain–Barré, polyradiculoneuropathy) because they were included in a single paper. The other words presented multiple connections, being present in more papers (Figure 1a).

Considering the classes, we found six groups of words and papers. The most populated was the group containing the terms stimulation, facial district, and femoral nerve, with 86 papers. The second group, in terms of numerosity, was the group containing polyneuropathies (immune-mediated and genetic), exercise, and electrotherapy. In the third group, trauma and injury were present, as were the terms immune, mobilization, and cryotherapy. The fourth group showed the terms diabetes and vibration therapy. In the fifth group, the other nerves were included together with compression and entrapment disease, orthosis, manipulation, and laser and ultrasound therapy. In the less populated group, we found the terms tumor and toxic in 17 papers (Figure 1b).

### 3.3. Relative Results of the Single Searches

The single searches combining the term rehabilitation with a nerve or a disease showed additional results (Table 1). The different rehabilitation approaches have been specially studied for entrapment neuropathies, which mainly involve upper limb nerves. For entrapment neuropathies, electromagnetic nerve stimulation provided good outcomes for symptom relief [39]. Among the other physical agents, laser therapy and ultrasound therapy have shown effectiveness in improving patients symptoms [40]. In entrapment neuropathies, neurodynamic exercises demonstrated good effectiveness [41]. Finally, different studies showed the utility of orthosis for symptom and function improvements [42]. In immune-related diseases, exercise and physical therapy have been studied, with positive results for patients’ wellness when combined with disease-specific drugs [43]. A single study assessed the biomodulation delivered by intravascular laser irradiation in patients with GBS, providing encouraging data for pain treatment [44]. Similar results for the effectiveness of exercise were visible in hereditary neuropathies, in particular in contrast to muscle weakness and fatigue [45]. Concerning the nerve tumors, a few papers were available, mainly for vestibular schwannoma and rehabilitation after the surgical removal of the tumor. In these patients, the rehabilitation was focused on balance improvement and hearing function. The few papers showed the usefulness of proper physical training and the application of neurostimulation to the vestibular system [46]. Among the toxic diseases, chemotherapy-induced neuropathies have been mainly evaluated, and exercises and physical therapies aimed at strengthening, endurance, balance, and sensory-motor function could be considered in ameliorating QoL [47]. In diabetic neuropathies, various forms of neural stimulation revealed utility for neuropathic pain, while exercise protocols showed importance for endurance and sensory-motor benefits [48]. In addition to these approaches (common with the other neuropathies), vibration therapy was evaluated, demonstrating improvement in pain, mobility, and sensation [49]. However, the general evidence level of these methods is low and moderate, and some results derive from pilot studies.

## 4. Discussion

**Figure 2:**
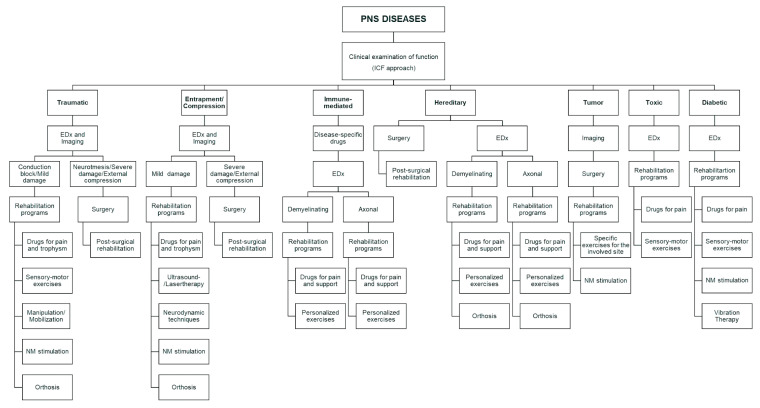
Flowchart summarizing rehabilitation approaches in different neuropathies. EDx—electrodiagnostic examination; NM—neuromuscular.

Our literature review shows the different approaches applicable to the different PNDs (Figure 2). In most cases, exercises and manual therapies are useful for patient management. They can involve the sensory-motor system in order to improve pain, sensory function, proprioception, and muscular function. The exercises are based on resistance and endurance, and, in general, a mixture of these can produce more benefits in a short-term evaluation (a few months) [50]. In literature, a real definition of the exercise sessions is not always available, but they should be administered considering the peculiarities of each patient and their needs. Usually, a personalized session duration and intensity are required. They are particularly important because they allow an adaptation of the patient for the energy requests and the oxygen consumption and a modification of muscle structure and function, which are fundamental for endurance [51]. Among the exercises, specific protocols exist for entrapment neuropathies. These exercises are based on nerve and tendon gliding, and they may reduce the compression on the nerve in a site [52]. In order to decide the most proper protocol, clinical evaluation should be based on the ICF method because the restoration/improvement of the function is the target of rehabilitation [34]. However, a clinical examination is not enough because the characterization of the nerve damage should be clear. The application of EDx should always be considered. If it has not been performed before the decision of the rehabilitation programs, it should be applied, especially because the decision between conservative or surgical can be based on its results and on clinical presentation. This is particularly evident in entrapment neuropathies, where severe conditions require surgery, in tumors, or in conditions where surgery is necessary for specific corrections [26]. Undoubtfully, rehabilitation can even intervene after the surgery to facilitate the recovery. Additionally, EDx allows the distinction between axonal and demyelinating diseases, and this information may support personalization of the rehabilitation treatment, focusing on specific impairments like strength, fatigue, and hypotrophy. Furthermore, imaging techniques are fundamental for guiding the treatment and avoiding side effects [53]. In cases of neurodynamic exercises, the physician should prescribe or perform this kind of evaluation to determine the exact site of the nerve suffering in cases of trauma or compression. EDx and imaging are also fundamental for the follow-up evaluation and extremely important in rehabilitation for monitoring the goals and objectives [54]. Physical agents represent the other large facet of rehabilitation. A lack of evidence exists for these treatments, but many authors showed promising results for ultrasound and laser therapy, even if they have been studied especially for compressions and entrapments. Although they are based on different kinds of energy, they can positively impact nerve and muscle function [55]. The other physical agent well studied is vibration therapy, which is useful for diabetic neuropathy. The rationale for its application is due to its capability to stimulate the sensory pathways, improving in this way pain sensation [56]. Pain is indeed one of the most common and severe symptoms of the different PNDs. For this reason, a complete rehabilitation project should include the use of drugs for neuropathic pain and, eventually, psychological support for the patients [57]. Pain modulation represents the main target of the nerve stimulations. They include TENS and peripheral magnetic stimulation. These can interfere with the mechanisms of pain generation and play an adjunctive role in symptom reduction in many neuropathies. Central nervous system stimulation is possible in PNDs, especially to treat chronic pain [58]. Neuromuscular electrical stimulation deserves a particular interest in traumatic nerve injuries because it can facilitate axonal growth and muscular preservation, but unclear evidence exists at the moment [59]. Finally, orthoses are useful in entrapment neuropathies for alleviating the symptoms, in traumatic injuries, and in hereditary neuropathies for preventing abnormalities or supporting diminished function [60].

Our literature review, however, despite the high incidence of PNDs, shows a relatively low number of papers exploring their rehabilitation. This is particularly evident for traumatic nerve lesions and toxic neuropathies. Additionally, only a limited number of types of nerve tumors are explored in the literature. This result should stimulate further and larger studies to define the best rehabilitation protocols. Another important limitation of the current literature is the relative inhomogeneity of the protocols: different types of exercises with several modalities exist, as do different types of neurostimulations [11].

Considering the methodology of lexical analysis, the inhomogeneity of the scientific interest of the different approaches and diseases is clearly visible. If diabetes and exercise are widely investigated, other common topics present very low frequencies. In particular, entrapments and traumas are scarcely represented. This result should be connected with the usual terms used to convey specific meanings. Being a lexical analysis, our analysis is determined by the words used in the different papers. This should encourage the use of the same terms to indicate the same significance across the world in order to facilitate the revision process and the data collection for generating evidence. Another utility of our lexical approach is the identification of families of words. In the graph, the strict association between diabetes and vibration therapy is well shown and confirmed by the following literature analysis. In a similar way, a close association between polyneuropathies and exercise is visible. This association is particularly interesting considering the high importance of exercise for diseases like CMT. Similarly, the nerves are strictly related to the terms entrapment and compression, which represent the most common forms of mononeuropathies. Hence, the approach can be used to facilitate an overview of the literature and depict the tendencies of the papers. Furthermore, the LENGTH approach, being based on the literature data extracted from the online database available elsewhere, is a reproducible method if the same search strategy and word identification are applied. The main limitation of the method is linked to the words themselves. The word list is decided on the basis of the authors’ experience, and the lexical analysis assesses the match of the words in the papers, thus only considering a limited number of associations and not considering all possible synonyms of the words [38].

Finally, our entire review shows some limitations. First, we have considered a large number of common neuropathies without covering all possible PNDs. Second, in our revision, we focused on families of treatments, avoiding a deep analysis of the single approaches. For example, we did not consider the different types of laser therapy or the different parameters of electromagnetic stimulation. Additionally, a detailed characterization of the single protocols for each disorder was out of the scope of this review, which focused on the general presentation of different PNDs and their possible rehabilitation.

## 5. Conclusions

Rehabilitation of PNDs is a complex field requiring extensive knowledge of the different neuropathies, their clinical presentation, and the methods to assess them, like EDx and imaging techniques. The protocols applicable to care for the patients are several, with different features and different aims. Drugs are used for pain management and to support patients’ functions; physical agents have a vast range of effectiveness, from pain sensation to muscle improvement; exercise aids sensory-motor restoration and the acquisition of useful strategies; and orthoses help reduce disabilities. PNDs may have different degrees of severity, but they usually negatively impact daily activity. Because of their complexity and impact, PNDs represent a real challenge for rehabilitation, which is sometimes the only effective treatment. Although the literature shows the usefulness of several approaches, the development of specific rehabilitation protocols is still ongoing, and further research is needed to identify the most appropriate treatments.

## Figures and Tables

**Table 1:** Results of the single searches performed for nerves and diseases.

		Most Frequent Words	
**rehabilitation teherapies**		**diseas** **e**	**nerve**
	physical agent	entrapment	ulnar
	physical therapy	toxic	peroneal
	exercise	toxic	femoral
	manipulation	entrapment	radial
	mobilization	entrapment	radial
	electrotherapy	diabetic	median
	lasertherapy	entrapment	median
	ultrasound therapy	entrapment	radial
	stimulation	diabetic	tibial
	vibration therapy	diabetic	
	orthosis	entrapment	peroneal
**nerves**	facial	stimulation; exercise; physical therapy	
	median	stimulation; mobilization; exercise	
	ulnar	stimulation; mobilization; exercise	
	radial	stimulation; mobilization; exercise	
	peroneal	stimulation; exercise; orthosis	
	tibial	stimulation; exercise; mobilization	
	sural	stimulation; exercise	
	femoral	stimulation; mobilization; exercise	
**disesases**	diabetic	exercise; stimulation; physical therapy	
	entrapment	orthosis; exercise; stimulation	
	hereditary	exercise; physical therapy; orthosis	
	immune	exercise; physical therapy	
	toxic	exercise; physical therapy	
	tumor	stimulation; exercise; physical therapy	

## Data Availability

The data are available in the Medline database.
